# Huperzine A Production and Acetylcholinesterase Inhibition by *Phlegmariurus taxifolius* Cell Suspension Culture: A Comparative Study in Flasks and an Airlift Bioreactor †

**DOI:** 10.3390/ph18030383

**Published:** 2025-03-08

**Authors:** Rocío del Carmen Pérez Aguilar, Talia Rodríguez Salgado, Olga Lidia Cruz-Miranda, Alexis Uriel Soto Díaz, Ariadna Zenil Rodríguez, Lamine Bensaddek, Christian Carreño-Campos, María Luisa Villarreal, Anabel Ortiz-Caltempa, Alexandre Toshirrico Cardoso-Taketa

**Affiliations:** 1 Centro de Investigación en Biotecnología, Universidad Autónoma del Estado de Morelos, Cuernavaca 62209, Mexico; rp.aguilar.biotec@gmail.com (R.d.C.P.A.); talis2204@gmail.com (T.R.S.); olgacumi@hotmail.com (O.L.C.-M.); uriel.soto.diaz@outlook.com (A.U.S.D.); ariadna.zenil@uaem.mx (A.Z.R.); chrcam12@gmail.com (C.C.-C.); luisav@uaem.mx (M.L.V.); 2 Ecologie et Dynamique des Systèmes Anthropisés (EDYSAN UMR 7058 CNRS-UPJV), Université de Picardie Jules Verne, 80000 Amiens, France; lamine.bensaddek@u-picardie.fr

**Keywords:** *Phlegmariurus taxifolius*, Lycopodiaceae, huperzine A, cell culture, 250 mL flasks, 2 L airlift bioreactor, scale up production, Alzheimer’s disease, acetylcholinesterase

## Abstract

**Background**: The callus cultures from the fronds of the lycophyte *Phlegmariurus taxifolius* produce the huperzine A (HupA) alkaloid, which is used in Alzheimer’s disease treatment. This study aimed to establish the growth kinetics and HupA production by the newly HupS21 cell line grown in 250 mL flasks and in a 2 L airlift bioreactor. **Methods**: Batch-type kinetics were carried out for 60 days in 250 mL flasks and for 20 days in a 2 L airlift bioreactor. Measurements of dry weight (DW), specific growth rate (μ), doubling time (dt), pH, carbohydrate consumption, and HupA quantification were performed. The acetylcholinesterase (AChE) inhibitory assay of the HupS21 alkaloidal extract was determined. **Results**: The 250 mL flasks kinetic reached a maximum cell growth of 8.17 g/L DW, with a μ of 0.045 day^−1^ and a dt of 15.40 days. The maximum HupA production was of 2.03 μg/g DW at day 45. In the 2 L airlift reactor, a maximum growth of 16.70 g/L DW, a μ of 0.062 day^−1^, a dt of 11.20 days, and HupA production of 2.48 μg/g DW at day 15 were obtained. The alkaloidal extract from the HupS21 cell line at 100 μg/mL showed an AChE inhibitory activity of 85.6 ± 1.27%. **Conclusions**: The airlift reactor outperformed the flask cultures in maximum cell growth, specific growth rate, doubling time, and HupA production. To our knowledge, this research is the first report on the establishment of suspension cell cultures of *P. taxifolius* in shaken flasks and in an airlift bioreactor, providing a foundation for scaling up HupA production for pharmaceutical use.

## 1. Introduction

Alzheimer’s disease, the most common form of dementia, is a progressive and irreversible neurodegenerative disorder characterized by memory loss and cognitive decline [1]. Symptoms develop gradually, worsening over time, and severely impact daily life. The disease compromises learning, language, and judgement, ultimately leading to neuronal death [2].

Certain secondary metabolites of plants, such as some alkaloids from specific species of lycopodes, e.g., *Huperzia serrata*, have great biological importance since they have the ability to inhibit the function of acetylcholinesterase. This inhibition raises the levels of acetylcholine at cholinergic synapses, acting on muscle contraction and neuronal transmission and signaling [3]. Huperzine A (HupA) is a drug used to treat this disease that acts as an inhibitor of the enzyme acetylcholinesterase [4]. Several studies in vitro and in vivo have demonstrated the neuroprotective activity of HupA. In addition, cognitive improvement has been demonstrated in patients who have been medicated with HupA. The molecule acts on various signaling pathways in both pre- and post-synaptic mechanisms. It also acts at the molecular level of proteins involved in neurological diseases, such as the amyloid precursor protein (APP) and the beta-amyloid peptide [5]. HupA is an alkaloid isolated mainly from the clubmoss *Huperzia serrata* that has attracted great attention for its efficacy and safety in the treatment of Alzheimer’s [5].

Overharvesting of *H. serrata* for HupA extraction has disrupted its ecological balance, leading to rapid decline populations [6]. In this sense, it is imperative to search for sustainable production methods of HupA, either by finding species that can be sustainably cultivated by employing biotechnological strategies such as in vitro plant culture or expressing the HupA pathway in heterologous systems [6]. Therefore, it is crucial to explore alternative lycopod species, such as the Mexican endemic *P. taxifolius* (Sm.) Löve and Löve, family Lycopodiaceae, as potential sources of HupA. Furthermore, *Phlegmariurus* species are among the epiphytic lycophytes that are categorized as threatened according to the International Union for Conservation of Nature (IUCN) criteria at the regional level in Mexico [7]. Therefore, biotechnological research on these species can support their conservation by reducing reliance on wild populations and promoting sustainable use. 

Our research group patented a method for callus induction from *P. taxifolius* fronds [8], and the establishment of in vitro callus cultures using a semisolid culture Gamborg B5 medium was achieved. The calluses were adapted in a liquid medium containing naphthaleneacetic acid (NAA) and zeatin (ZT), both at 0.5 mg/L, affording the HupS21 cell line that produces 0.53 μg/g DW of HupA, in contrast to the value of 265 μg/g DW found in the wild plant’s fronds.

This study is the first to explore HupA production in *P. taxifolius* cell suspension using flasks and an airlift-type bioreactor. We optimized the HupA production using in vitro cultures of *P. taxifolius*, contributing to sustainable and scalable production methods.

## 2. Results

### 2.1. Cell Suspension Cultures of Line HupS21

**Figure 1:**
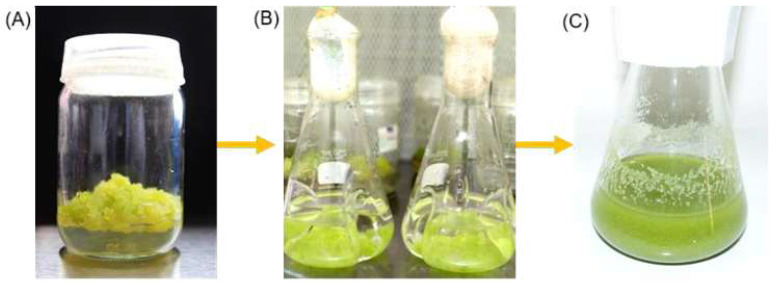
Establishment of the liquid suspension culture cells of *P. taxifolius* HupS21 line: (**A**) grown of friable calli on solid media; (**B**) calli grown in liquid media in three-baffled flasks; and (**C**) cell suspension culture.

The establishment of cell suspension culture of *P. taxifolius* line HupS21 started with the inoculation of friable callus in flasks with lateral baffles containing liquid Gamborg B5 medium supplemented with plant growth regulators (PGRs) NAA (0.5 mg/L) and ZT (0.5 mg/L). Once the complete disaggregation of the calluses was observed, they were transferred into Erlenmeyer flasks (Figure 1).

**Figure 2:**
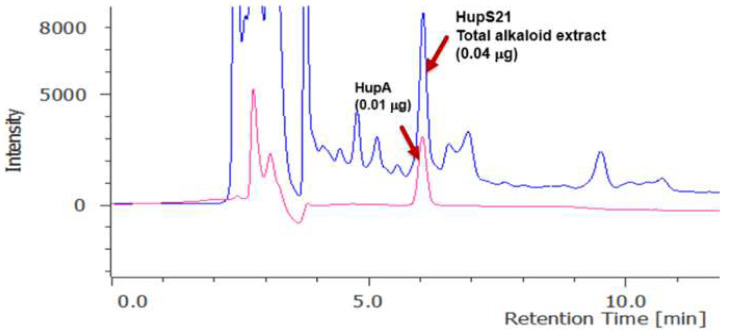
HPLC profiling of the HupS21 total alkaloid extract (blue line) and the huperzine A standard (red line). Chromatographic conditions: C18 column, mobile phase of H_2_O/TFA (0.0125%):CH_3_CN (85:15), at 1 mL/min, and detection at 310 nm.

To verify that the *P. taxifolius* suspension produced HupA, the analysis of its alkaloid extract obtained from a 40-day HupS21 suspension was carried out by high-performance liquid chromatography (HPLC). The presence of a peak at 6.1 min coincided with the retention time of the HupA standard (Figure 2).

### 2.2. Kinetic Study of the Suspension Cells of the HupS21 Line in Flask

**Figure 3:**
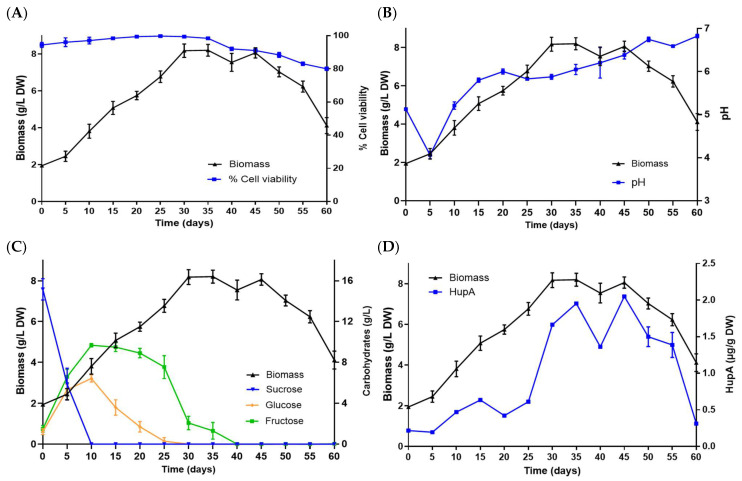
Kinetic study and production of HupA in cell suspension culture of *P. taxifolius* HupS21 line grown in 250 mL flask: (**A**) cell viability; (**B**) pH; (**C**) carbohydrate consumption; (**D**) HupA production. HupA production. Bars represent ±SD (n = 3). See Table S1: R^2^ biomass vs. depletion of carbon source; and Table S2: R^2^ biomass vs. HupA production.

The HupS21 cell line cultured in shake flasks showed a growth pattern with a short lag phase between days 0 and 5. The cells started the exponential phase after 5 days in culture until day 30. Maximum growth was achieved at 30 days, reaching an average biomass of 8.17 ± 0.03 g/L DW that represented 4.1 times the initial inoculum of 10% *w*/*v* (100 g fresh weight; FW = 2 g DW). The cell division parameters with µ = 0.045 day^−1^ and dt = 15.40 days were recorded once the cells entered the stationary phase (day 31). Even though after the completion of carbohydrate uptake at day 45, the cell viability remained high (95%) and started to decline after the stationary phase, with values of 70% at day 60. Sucrose was completely converted into fructose and glucose at day 10. Glucose was totally consumed at day 30, whereas fructose was available until 40 days of culture. The pH variation initially decreased shortly after inoculation of cells into the culture to values of 4 and remained in the range of 5 to 6.8 until day 60. Concerning the HupA production, the yield of this alkaloid was 2.03 μg/g DW on day 45, achieving an increase of 4.83 times with respect to the initial concentration (0.42 μg/g DW). The second point of high HupA production was on day 35 with 1.97 μg/g DW. However, on day 40, there was a drop in alkaloid production (1.30 μg/g DW), which coincided with a decrease in biomass. HupA is a metabolite associated with growth since as the biomass increases, there is an increase in this compound (Figure 3)**.**

### 2.3. Kinetic Study and HupA Production of Airlift Bioreactor-Grown Cell Suspension Culture of P. taxifolius

**Figure 4:**
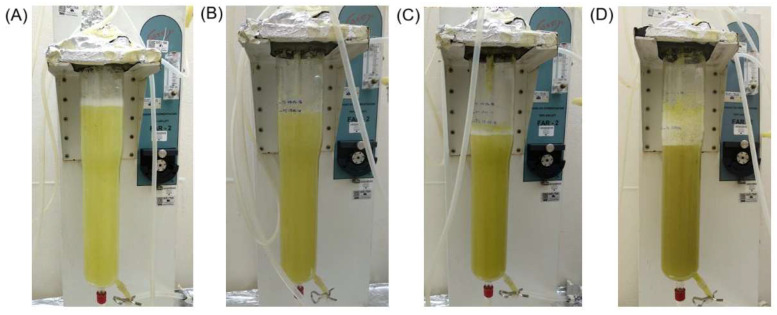
HupS21 line scale-up in 2 L airlift bioreactor: (**A**) day 0; (**B**) day 10; (**C**) day 15; and (**D**) day 20. The medium B5 was supplemented with NAA (0.5 mg/L), ZT (0.5 mg/L), CW (20 mL/L), sucrose (20 g/L), polyvinylpyrrolidone (PVP) at 1 g/L, and potassium nitrate at 5 g/L.

The HupS21 line was scaled up from 250 mL flask to a 2 L airlift bioreactor in order to increase HupA production, starting from a 10% (*w*/*v*) inoculum. A 20-day kinetics run was performed, and the same parameters as in the flask kinetics were measured. Figure 4 shows the growth of the HupS21 culture in the 2 L airlift bioreactor, with pictures taken at days 0, 10, 15, and 20.

**Figure 5:**
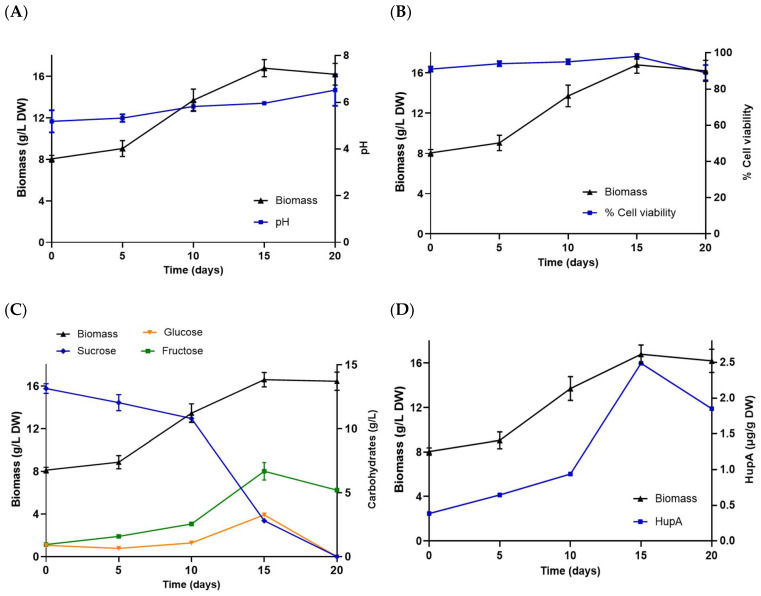
Kinetic study and production of HupA in cell culture of *P. taxifolius* HupS21 line grown in 2-L airlift bioreactor: (**A**) cell viability; (**B**) pH; (**C**) carbohydrate consumption; (**D**) HupA production. Bars represent ± SD (n = 3). See Table S3: R^2^ biomass vs. depletion of carbon source; and Table S4: R^2^ biomass vs. HupA production.

The *P. taxifolius* cell line HupS21 in the bioreactor experienced an adaptation phase lasting 5 days. Then, an exponential phase reached the highest production of biomass on day 15 with 16.70 ± 0.08 g/L DW, which is 2.09 times the size of the initial inoculum of 10% *w/v* (180 g/L FW = 8 g DW, for a volume of 1.8 L), and a growth rate of μ = 0.062 day^−1^ and a dt = 11.20 days. A 2.5-fold increase in biomass production compared to shake flask cultures was observed, which was maintained until day 20 before experiencing a gradual decrease. The cell viability of the culture was 91% to 98.5% at the end of its cell growth. The pH presented slight variations during cell growth, starting at 5.18 to reach a maximum of 6.52 on day 20. The carbon source underwent complete hydrolysis, with a preference for consuming glucose until it is depleted on day 20. Figure 5 shows that the production of HupA was directly linked to the rise of biomass in the culture, reaching a peak of 2.48 µg/g DW on day 15. This maximum HupA production culminated at the end of the exponential growth of the culture.

A summary of the key parameters recorded in the kinetics of HupS21 cultures in flasks and in a bioreactor can be seen in Table 1.

### 2.4. In Vitro Acetylcholinesterase Inhibitory Activity

The acetylcholinesterase activity of the alkaloid extract of the HupS21 suspension cells at 100 μg/mL presented an enzyme inhibition of 85.6% ± 1.27. HupA was used as a positive control at 0.16 μg/mL and displayed an acetylcholinesterase inhibitory value of 48.3% ± 0.28, which was in accordance with the previous IC_50_ reported [9].

## 3. Discussion

The callus-derived cell suspension line HupS21 has been continuously cultured over a three-year period in our laboratory, using culture medium B5 added with naphthaleneacetic acid (NAA) and zeatin (ZT). Prior to initiating the kinetics in batch and bioreactor cultures of HupS21, the suspension-derived callus line was confirmed as HupA producer, thereby serving as inoculum for growth kinetics. Not only did the HupS21 line produce HupA, but its alkaloid extracts also showed anticholinesterase activity, with an acetylcholinesterase inhibition of 85.6% at 100 μg/mL, showing that this species has an interesting potential as a source of compounds with anticholinesterase action for the Alzheimer’s treatment. This result is very promising when compared with literature data; e.g., the hydroalcoholic extract of adventitious root cultivated by in vitro culture of *Mondia whitei* (Hook.f.). Skeels showed acetylcholinesterase inhibition in adventitious roots with an IC_50_ of 2.13 mg/mL [10]. As can be appreciated in Figure 2, the total alkaloid extract of HupS21 presents other compounds that could be other alkaloids, but this assumption needs to be confirmed in the future with more research.

HupS21 cell cultures presented high viability, while the carbohydrate source remained available until day 45, reaching 95%, and then decreased to 70% at the conclusion of the kinetic study on day 60. The high cell viability at the end of the kinetics can be explained because the cultures remained green, so the cells carry out photosynthesis and can produce energy and carbon sources. Day 45 coincided with the maximum HupA production. In light of this, it would be intriguing to implement a fed-batch culture strategy, which involves introducing carbohydrates into the culture medium during the growth process. This procedure could stimulate HupA production during the stationary phase of growth.

The cut-off point for the experiments in flasks was 60 days, in contrast to 20 days for the experiments in the bioreactor. This is because the airlift bioreactor has many benefits over flask-based cultures. For example, it has better mixing and aeration factors, which help with mass and heat transfer, and it also has less mechanical stress, which improves cell growth [11].

The results showed that the growth rate of HupS21 cells cultured in shaken flasks was relatively slow, with a dt = 15.4 days, when compared with the mostly cultured cells, but slightly faster compared to the *Taxus cuspidata* (dt = 20 days). Its taxol accumulation up to 4.0 μg/g DW extract [12] has a similar magnitude level as HupA produced (2.03 μg/g DW) by the HupS21 cell line. Using the same batch culture method in shaken flasks, both cultures grew less quickly than the batch culture of seed plants, e.g., *Galphimia glauca*, which had a dt of 4.3 days, which is typical for most plant cell suspension cultures [13,14].

The growth rate of the HupS21 cell line cultured in the bioreactor was μ = 0.062 day^−1^, with a dt = 11.20 days, which contrasts with µ = 0.045 day^−1^ and dt = 15.40 days values for the suspended cells cultured in flasks. These results indicate that the growth rates in the bioreactor were superior to those of the batch cultures, representing a 204.4% increase in the maximum biomass production. Furthermore, it is noteworthy to note that the bioreactor achieves maximum HupA production at day 15 (2.48 μg/g DW), compared to day 45 in the flasks (2.03 μg/g DW). This suggests that the technology using the airlift system offers advantages, possibly due to higher oxygenation and better nutrient distribution. The regression coefficient (R^2^) analysis of biomass vs. depletion of carbon source and biomass vs. HupA production, with R^2^ > 0.7 and *p* < 0.05, for both the flasks and bioreactor kinetics data (see Tables S1–S4), indicated positive correlations between biomass production, sugar consumption, and HupA production.

When comparing the growth parameters in a 2 L airlift bioreactor of cell cultures of *P. taxifolius* with those of *Ageratina pichinchensis* (Kunth) R.King and Ho.Rob, Asteraceae, it is clearly noticeable that the clubmoss species are much slower growing, since the kinetics parameters for the cell culture of *A. pichinchensis* were µ = 0.2216 day^−1^ and dt = 3.13 days [15].

The 2 L airlift bioreactor culture only afforded 1.2 times more HupA than flasks, which is still insufficient to be considered a viable production alternative. However, this biotechnological strategy has many benefits, e.g., growth control and reproducibility, including higher biomass production. The present approach of cultivating the HupS21 cell line in a bioreactor has a lot of potential for industrial scaling to meet the needs of the pharmaceutical industry, since the current production of HupA is based on the direct extraction of the *Huperzia serrata* plant [16]. Similarly, the problems surrounding the production of taxol from *Taxus* spp., like its low yield in wild plants (0.02% DW), their overexploitation, and their long growth times, are issues shared with *P. taxifolius*. Then, the elicitation and stress exposures, as well as two-phase processes [17], are different approaches applied to increase the taxol synthesis using in vitro culture of *Taxus* species that could serve as a model for research that aims to boost HupA production from lycophytes.

Among the strategies we have implemented in our laboratory to have a HupA-producing culture system, we can mention the isolation of an endophytic fungus from the fronds of *P. taxifolius* [18]. A fungal isolate from the *Fusarium* genus cultured for 14 days yielded 3.2 μg/g Hup A in mycelial dry weight, which is 1.3 times higher than maximum production in the bioreactor culturing HupS21 cells at day 15 (2.48 μg/g dry weight). However, the genetic and phenotypic instability of endophytic fungi cultures is well known, which is a drawback compared to plant-based fermentations [19].

Furthermore, the current study utilized a 2 L bioreactor with a cultivation period of 20 days, a time that differs from that of clubmoss species in nature; e.g., *Huperzia serrata*’s life cycles require 15 years to develop from the spore form to the sporophyte stage, which is the vegetative form used for commercial exploitation. In addition, clubmoss species need very specific ecological niches to grow, making their agronomical approach a challenge [20]. This is why cell cultures may be advantageous for the conservation of this species, as they reduce the need for collecting in the wild.

## 4. Materials and Methods

The solvents used for HPLC analysis were of analytical grade and HPLC grade, J.T. Baker (Avantor, Radnor, PA, USA). A Symmetry C18 column was used for HPLC studies on an analytical scale (Waters, Milford, MA, USA; 5 μm, 4.6 mm I.D. × 250 mm length). Chemicals for bioassays and (−)-huperzine A standard were purchased from Sigma-Aldrich (St. Louis, MO, USA).

### 4.1. Callus Growth in Semisolid Medium

Calluses of the HupS21 line of *P. taxifolius*, previously obtained from callogenesis induction of plant fronds [8], were grown in Gamborg (B5) medium supplemented with naphthaleneacetic acid (NAA) at 0.5 mg/L combined with zeatin (ZT) at 0.5 mg/L, coconut water (CW) at 20 mL/L, sucrose (20 g/L), polyvinylpyrrolidone (PVP) at 1 g/L, potassium nitrate at 5 g/L, and phytagel (3.5 g/L); pH was adjusted to a value of 5.5 ± 0.2 using a pH meter, and the medium was then autoclaved at 121 °C for 15 min. Cell cultures were grown at 25 ± 2 °C under photoperiod conditions of 16 h light/8 h darkness. The luminous intensity in the culture room was 25 μmol/m^−2^ s^−1^ [8,21].

### 4.2. Establishment of Cell Suspension Cultures in Shake Flasks

To establish the cell suspension culture of the line HupS21, 10 g of fresh weight (FW) friable calli were added into 250 mL Erlenmeyer baffled flasks with 100 mL of culture medium B5 supplemented with NAA (0.5 mg/L), ZT (0.5 mg/L), CW (20 mL/L), sucrose (20 g/L), polyvinylpyrrolidone (PVP) at 1 g/L, and potassium nitrate at 5 g/L. The flasks were covered using cotton plugs. The cell suspensions were subcultured every 4 weeks by transferring 10% *w*/*v* (10 g/100 mL) inoculum into 250 mL fresh medium. These cultures have been maintained for more than six years in our laboratory under the conditions described. Flasks were maintained at 25 ± 2 °C in an orbital shaking incubator (AGO-6090 equipment) operating at 115 rpm in continuous light (50 μmol/m^−2^ s^−1^). For kinetic studies, 25-day cultures of the cell line were inoculated at a 2% *w*/*v* in 100 mL of B5 liquid medium using 250 mL Erlenmeyer flasks.

### 4.3. Kinetic Studies of Cell Suspension Cultures in Shake Flasks

The cultures of *P. taxifolius* cell line HupS21 were evaluated for a period of 60 days, taking samples every 5 days in triplicate, and the following parameters were registered: fresh weight, dry weight, cell viability, pH, carbohydrate consumption, and HupA concentration. The samples were subjected to freeze-drying at −53 °C for 120 h (LABCONCO equipment), and cell dry weight was recorded. Cell viability was determined by the fluorescein diacetate (FDA) method [22]. Carbohydrate consumption (sucrose, glucose, and fructose) was estimated by determining their concentrations in the culture medium by the HPLC method (Waters 7117 plus Autosampler, Milford, MA, USA). UG80 amino column, size 5 μm (4.6 mm × 150 mm), purchased from Osaka Soda Co., Osaka, Japan, was used. The HPLC conditions were as follows: mobile phase, 80:20 acetonitrile–water; flow rate, 1 mL/min; temperature, 25 °C [23].

### 4.4. Cell Suspension Cultures of Cell Line HupS21 in Airlift Bioreactor

To scale up suspension cultures of the cell line HupS21, a 2 L custom-made airlift bioreactor was used. The parameters of the airlift bioreactor were the following: 52 cm in height and 7 cm in diameter, with a 27 cm draft tube height, a 2.7 cm inner draft tube diameter, and a 2.0 cm bottom clearance. The dynamic process of the bioreactor was achieved by spraying air from the bottom of the draft. This creates a stream of bubbles that move up and down, with the medium returning to the bottom after rising [23]. An industrial autoclave (SEV equipment) has been used to sterilize the airlift bioreactor. After that it was filled with autoclaved B5 medium (1.8 L), supplemented with NAA (0.5 mg/L), ZT (0.5 mg/L), CW (20 mL/L), and sucrose (20 g/L). The HupS21 suspension cell line was an appropriate candidate for scale-up in a bioreactor since it responded well in flask cultures. An inoculum of 10% FW (*w*/*v*) from a 15-day-old HupS21 culture grown in 1 L Erlenmeyer flasks containing 500 mL of B5 medium was used as starter material for the bioreactor kinetics. The 2 L bioreactor was incubated at 25 ± 2 °C under continuous light, using white light of 50 μmol/m^−2^ s^−1^, and operated at 0.5 vvm for 15 days and then at 1.0 vvm until the end of the culture period (20 days). Under these conditions, an adequate mixing of the cell suspension was achieved. Antifoam (Dow Corning Co.., Midland, MI, USA) at 0.1% *v*/*v* was added. The culture was sampled every 5 days to measure biomass, pH, carbohydrates content, and HupA.

Every five days, the kinetics underwent sampling under sterile conditions. For each sampling, 20 mL of medium was collected and filtered in Whatman N°2 filter paper to determine growth parameters and HupA production. The experiments were performed in triplicate.

### 4.5. Growth Kinetics

Biomass from shake flasks and bioreactor served as material to build the growth curves from which the specific growth rate (µ) was calculated from the slope of the straight line obtained by plotting the ln of the change in biomass concentration per unit time/initial biomass concentration (d^−1^) during the exponential growth phase. Doubling time (dt) was calculated using the equation dt = ln 2/μ [24,25].

### 4.6. Identification and Quantification of HupA

The identification and quantification of HupA were performed by HPLC. The dried and ground biomass, using a mortar, was degreased with hexane in a 1:3 ratio (*v*/*v*). A methanolic extract was prepared by soaking the degreased biomass in methanol at a 1:3 (*v*/*v*) ratio for 12 h and then sonicated for 5 min. This process was performed in triplicate. Finally, the total alkaloid extracts were prepared from the methanolic extract employing the acid-base extraction protocol [18]. For HPLC analyisis a JASCO equipment, model LCNet II/ADC, using a C18 column Shiseido CAPCELL PAK MG II S5 and a mobile phase composed of H_2_O/TFA (0.0125%):CH_3_CN (85:15), under a flow rate of 1 mL/min at 25 °C, was used. Analyte detection was at 310 nm. The standard curve of HupA at concentrations between 0.1 and 50 µg/mL was used for quantification. The parameters of resolution of Rs > 2 and linearity of R^2^ = 0.9955 indicated that the method used was valid and reliable [18].

### 4.7. Inhibitory Activity of the AChE

For the determination of the inhibitory activity of the acetylcholinesterase, the purified electric eel enzyme was used at a concentration of 0.02 U/mL (EC 3.1.1.7 Type VI-S, Sigma) [26]. The enzyme was incubated in 100 mM phosphate buffer (pH 8) with 10 μL of 5,5′-ditiobis-2-nitrobenzoic acid (DTNB, 0.5 mM; Sigma-Aldrich) and 10 μL of alkaloid extract from flask cultures at day 45, diluted in methanol (100 μg/mL). The plate was placed in a water bath at 25 °C for 30 min. Subsequently, 10 μL of acetylcholine iodide (20 mM) was added to each well to initiate the reaction. Hydrolysis was monitored by the formation of the anion 2-nitro-5-mercaptobenzoate, whose color is yellow, and its absorption spectrum is 412 nm. The kinetics of the reaction were recorded every 30 s for 3 min on the spectrophotometer with a microplate reader (SpectraMax ID3 reader; Molecular Devices, LLC, San Jose, CA, USA). Enzyme activity was calculated by comparing the activity of the non-inhibitor reaction. HupA standard was used as a positive control (0.16 μg/mL). The percentage of inhibition was calculated as % inhibition = (1 − S/E) × 100, where E and S are the activities with and without the inhibitor, respectively [18,26].

### 4.8. Statistical Analysis

Data of in vitro cultures are presented as the means ± standard deviations of three independent replicates and were analyzed by ANOVA and Tukey tests.

The inhibitory activity of the acetylcholinesterase was performed with three replicates. Means ± SD values were calculated, and the data were subjected to ANOVA followed by Dunnett’s test. Also, the Tukey’s test for multiple comparisons of the means was carried out to find differences at *p* < 0.05 between treatments. The statistical analysis was performed on the SAS 9.5 program.

A simple regression analysis was performed to determine the degree of correlation between dry weight biomass and carbon source depletion and HupA yield of plant culture in both the flask and the bioreactor, followed by calculating the coefficient of determination (R^2^). These analyses were carried out in the GraphPad Prism program (version 10.4.1).

## 5. Conclusions

From calluses of *Phlegmariurus taxifolius*, it was possible to establish cell suspension cultures named HupS21. The suspension cultures were grown in 250 mL Erlenmeyer flasks and then scaled up in a 2 L airlift bioreactor and produced huperzine A. The kinetic parameters of the two types of cultures were obtained and compared, which showed the advantages of cultivation in an airlift-type bioreactor. In the airlift bioreactor the accumulation of huperzine A was improved. This is the first time reporting the production of huperzine A from *P. taxifolius* in suspension cultures. The prospects of this research work include scaling up the process to higher-capacity bioreactors, along with the use of elicitors and metabolic engineering strategies to increase HupA production.

## Figures and Tables

**Table 1:** Kinetic parameters from 250 mL flasks and 2 L bioreactor cultures.

Kinetic Parameters	250 mL Flasks Culture	2 L Bioreactor Culture
Day of maximum growth	30	15
Maximum biomass production	8.17 ± 0.03 g/L DW	16.70 ± 0.08 g/L DW
Growth rate (µ)	0.045 day^−1^	0.062 day^−1^
Duplication time (dt)	15.40 days	11.20 days
Day of maximum HupA production	45	15
Maximum HupA production	2.03 μg/g DW	2.48 μg/g DW

## Data Availability

The data presented in this study are available on request from the corresponding authors.

## Supplementary Materials

The following supporting information can be downloaded at: https://www.mdpi.com/article/10.3390/ph18030383/s1, Table S1: Regression coefficient of biomass production vs. depletion of carbon source in the kinetic study of the flask-grown cell suspension culture of *P. taxifolius*; Table S2: Regression coefficient of biomass vs. Hup A production in the kinetic study of the flask-grown cell suspension culture of *P. taxifolius*; Table S3: Regression coefficient of biomass production vs. depletion of carbon source in the kinetic study of the airlift bioreactor-grown cell suspension culture of *P. taxifolius*; Table S4: Regression coefficient of biomass vs. Hup A production in the kinetic study of the airlift bioreactor-grown cell suspension culture of *P. taxifolius.*
