# Supplementary material for: Huperzine A Production and Acetylcholinesterase Inhibition by Phlegmariurus taxifolius Cell Suspension Culture: A Comparative Study in Flasks and an Airlift Bioreactor
Source: Pharmaceuticals (Basel). 2025 Mar 8;18(3):383. doi: 10.3390/ph18030383 (PMC11946177; doi:10.3390/ph18030383)
Supplement: Supplementary file 1 [file pharmaceuticals-18-00383-s001.zip › pharmaceuticals-3457630-supplementary.pdf]

Huperzine A Production and Acetylcholinesterase Inhibition by *Phlegmariurus taxifolius* Cell Suspension Culture: A Comparative Study in Flasks and an Airlift Bioreactor

Rocío del Carmen Pérez Aguilar[1], Talia Rodríguez Salgado[1], Olga Lidia Cruz-Miranda[1], Alexis Uriel Soto Díaz[1], Ariadna Zenil Rodríguez[1], Lamine Bensaddek[2], Christian Carreño-Campos[1], María Luisa Villarreal [1], Anabel Ortiz Caltempa[1*], and Alexandre Toshirrico Cardoso-Taketa [1*]

Supplementary Materials

Tables S1 – S4

Table S1. Regression coefficient of **biomass production vs. depletion of carbon source** in the kinetic study of the flask-grown cell suspension culture of *P. taxifolius.*

	Biomass	Fructose	Glucose	Sucrose
R squared (R^2)	0.8357	0.8462	0.8294	0.8802
P value	0.0001	0.0006	0.0004	0.0002
Deviation from zero?	Significant	Significant	Significant	Significant

Table S2. Regression coefficient of **biomass vs. Hup A production** in the kinetic study of the flask-grown cell suspension culture of *P. taxifolius.*

	Hup A
R squared (R^2)	0.7181
P value	0.0003
Deviation from zero?	Significant

Table S3. Regression coefficient of **biomass production vs. depletion of carbon source** in the kinetic study of the airlift bioreactor-grown cell suspension culture of *P. taxifolius.*

	Biomass	Fructose	Glucose	Sucrose
R squared (R^2)	0.8660	0.8883	0.9140	0.8857
P value	<0.0001	<0.0001	<0.0110	<0.0170
Deviation from zero?	Significant	Significant	Significant	Significant

Table S4. Regression coefficient of **biomass vs. Hup A production** in the kinetic study of the airlift bioreactor-grown cell suspension culture of *P. taxifolius.*

	Hup A
R squared (R^2)	0.8294
P value	0.0316
Deviation from zero?	Significant
